# Training and delivery of a novel fatigue intervention: a qualitative study of rheumatology health-care professionals’ experiences

**DOI:** 10.1093/rap/rkz032

**Published:** 2019-08-27

**Authors:** Emma Dures, Clive Rooke, Alison Hammond, Sarah Hewlett

**Affiliations:** 1 Department of Nursing and Midwifery, University of the West of England; 2 Academic Rheumatology, Bristol Royal Infirmary, Bristol; 3 Centre for Health Sciences Research, School of Health and Society, University of Salford, Manchester, UK

**Keywords:** qualitative, fatigue, self-management, skills training, cognitive-behavioural, rheumatology, health-care professionals

## Abstract

**Objectives:**

Successful, non-pharmacological research interventions are challenging to implement in clinical practice. The aim of the study was to understand the experiences of rheumatology nurses and occupational therapists (tutors) delivering a novel fatigue intervention in a trial setting, and their views on requirements for clinical implementation. After training, tutors delivered courses of a manualized group cognitive-behavioural intervention to patients with RA in a seven-centre randomized controlled trial [Reducing Arthritis Fatigue by clinical Teams using cognitive-behavioural approaches (RAFT)], which demonstrated reduced fatigue impact at 2 years.

**Methods:**

Fourteen tutors participated in interviews, and eight tutors also participated in a focus group. Data were audio-recorded, transcribed and analysed using inductive thematic analysis.

**Results:**

The following five main themes were identified: ‘exciting but daunting’ reflected the mixture of excitement and anxiety in intervention training and delivery; ‘skills practice and demonstrations were essential’ captured the value of learning and practising together, even though the process could be uncomfortable; ‘an individual approach to a standardized intervention’ showed how tutors negotiated adherence to the manual with delivery using their own words; ‘becoming a better practitioner’ described how participation enhanced tutors’ wider clinical practice; and ‘pragmatic and flexible’ highlighted practical adaptations to facilitate training and intervention roll out.

**Conclusion:**

These insights inform strategies for clinical implementation of an evidence-based intervention that addresses a patient priority, with implications for other successful research interventions. Tutors believed that the skills acquired during RAFT enhanced their wider clinical practice, which highlights the benefits of upskilling members of clinical teams to provide self-management support to patients.

**Key messages**

- Evidence from trials of complex interventions often fails to translate into changes in clinical practice.
- The study findings inform strategies for clinical implementation of a novel intervention that addresses a patient priority.
- Upskilling members of clinical teams to provide self-management support to patients enhances their wider clinical practice.

## Introduction

Fatigue is a common and distressing symptom in inflammatory rheumatic diseases [1]. An international study of >6000 patients found that half were severely fatigued, defined as scoring ≤ 35 on the SF-36 vitality scale [2]. Research in RA has established that fatigue is present on most days for most patients, with >70% reporting levels similar to chronic fatigue syndrome [3, 4]. A survey of >1200 patients with inflammatory rheumatic diseases in England found that 82% wanted support to manage the impact of pain and fatigue [5]. Although rheumatology health-care professionals recognize the importance of fatigue, they do not know how best to help patients to deal with it [6, 7]. Although cognitive-behavioural therapy interventions have improved RA fatigue [8–11], implementation in clinical practice remains a challenge [12]. It has been suggested that much health-care research is wasted because publications of trials focus on the results and fail to describe interventions adequately [13]. Qualitative research can help by providing insights into factors that influence implementation [14].

RAFT (‘Reducing Arthritis Fatigue – clinical Teams using cognitive-behavioural approaches’) is a randomized controlled trial of a manualized, group-based self-management intervention to reduce the impact of fatigue [15]. The intervention comprises six sessions of two hours, held weekly, with a seventh, one hour long consolidation session held 8 weeks later. It is based on cognitive-behavioural approaches (CBA), including guided discovery, daily activity diaries and goal setting, in addition to supported peer learning in a group. Cognitive-behavioural approaches promote a shift in beliefs and progressive adaptations in how patients cope with fatigue, leading to better knowledge, confidence and reactivation in everyday activities [16–18].

The intervention is designed to be co-delivered by rheumatology health-care professionals (tutors) in pairs after training (see Supplementary Materials, available at *Rheumatology Advances in Practice* online). In RAFT, the tutors were nurses and occupational therapists (OTs). Their training comprised two parts: 4 days face-to-face training delivered centrally by a clinical psychologist and specialist OT (trainers), and incorporating CBA, managing group dynamics and the manual content; and observation by the trainers of each tutor pair delivering the intervention to patients, in a practise run.

During RAFT, a selection of sessions was observed by an independent clinical psychologist to ensure intervention fidelity. The intervention reduced fatigue impact at 26 weeks, with the benefits maintained at 2 years [8].

The aims of the present study were to understand tutors’ experiences of intervention training and delivery and to collect views on potential implementation of the intervention.

## Methods

The study was approved by the East of England – Cambridgeshire and Hertfordshire Research Ethics Committee (reference: 13/EE/0310). There were two methods of data collection: individual interviews to explore each tutor’s viewpoint and enable discussion of sensitive topics [19]; and a focus group to facilitate discussion and reflection of a common experience [20].

### Sample

All 15 tutors who delivered intervention sessions at the seven participating sites were invited to take part in an interview and a focus group (see Supplementary Materials, available at *Rheumatology Advances in Practice* online).

Fourteen tutors (nine nurses and five OTs) participated in an interview. Eight tutors (three nurses and five OTs) also participated in the focus group. At least one tutor from five of the seven RAFT sites participated in the focus group. Tutors were Band 6 or 7 (i.e. in senior roles) and had been qualified for a mean 18.3 years, with 5.5 years rheumatology experience (ranges 6–30 and 0–17 years); 10 had some experience of delivering information sessions to patient groups as part of patient education programmes, and three had prior knowledge of cognitive-behavioural therapy or goal-setting (13 of 14 tutors provided data).

### Data collection

Interviews were conducted face-to-face by E.D. at the tutors’ local hospitals where they had delivered the intervention. Data were collected between 2 weeks and 2 months after tutors had completed intervention delivery. The focus group was held in the Southwest of England, facilitated by E.D. and S.H. Before the start of the interviews and focus group, each tutor signed a consent form. Interviews were audio-recorded and lasted between 52 and 82 min (average 62 min). The focus group was audio-recorded and lasted for 84 min.

### Data analysis

Interview and focus group audio-recordings were transcribed, checked for accuracy against the original audio-recordings and anonymized. The interview and focus group transcripts were analysed using a data-driven, inductive thematic approach with no *a priori* theory or framework applied to the data [21, 22]. E.D. read all transcripts and coded chunks of text that related to the research topic. Related clusters of coded text formed sub-themes, which were grouped together to form a smaller number of higher-order themes that described broad, often abstract, elements in the dataset. Two interview transcripts were independently coded by S.H. and A.H., and a single interview transcript by C.R. The focus group transcript was analysed by E.D. and A.H. Owing to the overlap in the topics discussed, the focus group themes were used as a form of triangulation and compared with the interview themes. The findings are presented as a single, integrated analysis. Each theme has three parts: the label; the summary; and the supporting sub-themes, supported by data excerpts that link interpretation to tutors’ words (see Table 1) [23]. Data excerpts are identified using arbitrarily allocated numbers not linked to RAFT site or tutor pairing (INT1–14 for interview participants, FG1–8 for focus group participants).

**Table 1:** Theme labels and supporting sub-themes

1. Exciting but daunting	A different way of working Putting in time and hard work Feeling challenged Harder than it looks
2. Skills practice and demonstrations were essential	Being new to RAFT together Learning from expert demonstrations Role play: invaluable despite the discomfort Delivery improved with trainer feedback
3. An individual approach to a standardized intervention	Personalizing the manual The dynamics of pair work
4. Becoming a better practitioner	Working with the whole person Knowing how to draw things out, sit back and listen Confident talking about fatigue
5. Pragmatic and flexible	Adapting training and support Buy-in from managers and clinical colleagues

RAFT: Reducing Arthritis Fatigue by clinical Teams using cognitive-behavioural approaches.

## Results

### Theme 1: exciting but daunting

Tutors started the face-to-face training without a clear idea of what to expect from RAFT. As the complexity of the intervention became apparent, training and delivery were experienced as exciting but daunting.

#### A different way of working

The intervention required a way of working which was ‘completely unfamiliar territory’ (INT4). The use of CBA often contrasted with tutors’ usual clinical practice, which involved giving advice and problem-solving for patients.

INT3: “I wasn’t used to that kind of role, the cognitive-behavioural role rather than, just, as a nurse you just want to help them and say ‘Yes, I’ll do that for you’. So it was changing my kind of way of thinking.”

FG1: ‘it’s the techniques because we’re used to giving information, aren’t we, and obviously we have to deliberately not do that and reflect things all the time and that wouldn’t come naturally to most practitioners.’

#### Putting in time and hard work

Initially, tutors had not anticipated the time and effort needed to become familiar and confident with a large amount of material, delivered using a new approach, to patients in a group setting.

INT6: “It wasn’t something you were just going to be able to go away with and think, ‘I could just do this’ … you really did need to know your material. You needed to be well read … before you delivered … and it made you realize that it, there was quite a lot of preparation to be done.”

#### Feeling challenged

Although ‘interested, enthusiastic, I just wanted to try and do it’ (INT12), tutors reported feeling challenged as they started to engage with the training and intervention.

INT1: ‘it was a bit scary, actually, and I felt quite challenged.’

INT7: ‘I did feel very daunted starting it. I was very nervous delivering the groups as it first began.’

#### Harder than it looks

When the trainers demonstrated sessions, the delivery flowed, and the interaction appeared effortless. When tutors practised, they found it difficult to replicate this. Initially, this diminished tutors’ confidence, but it also gave them a standard to aim for.

INT3: ‘I didn’t feel particularly confident in delivering it as well because you’ve got professionals who have shown us how to do it, they’re so good at what they do.’

FG2: “[trainers] made it look so simple, so easy. I just think, ‘Why can’t I do it like that?’.”

### Theme 2: skills practice and demonstrations were essential

The opportunity to train with other nurses and OTs was valued. It was acknowledged that role play was essential, even though this could be uncomfortable.

#### Being new to RAFT together

Although skills practice was challenging, tutors were encouraged by knowing that they were all new to the intervention and were supporting each other to learn.

INT4: ‘It’s reassuring, in a way, to know that you have people with the same mind-set and obviously finding that opportunity to explore this territory further helps us because it’s like putting a blank canvas and at the end of the day it’s like painting it together.’

#### Learning from expert demonstrations

Observing the trainers demonstrate sessions brought the intervention to life, adding depth and detail to the manual.

INT7: ‘they demonstrated how to use the CB approach in the group setting using the manual; that was good because I work best by being able to observe.’

#### Role play: invaluable despite the discomfort

Tutors acknowledged role play as one of the most useful aspects of the training. Practising sessions out loud, then getting feedback from the trainers was invaluable, even though it could be difficult.

INT8: ‘For me to actually have a go and to practise … I found that was very useful even though I didn’t like it; I would say, one of the most valuable things of the course.’

#### Delivery improved with trainer feedback

Tutors reported that ‘you need the feedback on your performance’ (INT13) when delivering to patients.

FG5: ‘as much as it was really scary to have someone observe you, the feedback and the debrief afterwards was really good to just give you focus, direction and just reassurance that you were doing something right.’

### Theme 3: an individual approach to a standardized intervention

The manual was ‘my bible all the way through’ (INT9), because it contained the information needed to deliver the intervention. However, tutors described a tension between adhering to the manual content *vs* using their own words to deliver the intervention in a natural manner.

#### Personalizing the manual

Tutors consolidated and deepened their understanding of the intervention through adapting their manual, including writing summaries and re-phrasing text.

INT2: ‘because you’re trying to use that cognitive behavioural approach, I was conscious that maybe if I alter it too much, I wouldn’t be doing that.’

FG2: ‘that was one of the hardest things I think we’ve found. Is actually putting it on our words because …’

FG1: ‘we really struggled to start with because we were trying to learn it as a script and it wasn’t how we would say things.’

#### The dynamics of pair work

Tutors developed ways of working with their co-tutor(s) that were mutually supportive and allowed them each to play to their strengths. The ways in which tutors worked together was an important aspect of their individual approach to standardized content.

INT10: ‘you’re helping each other out, aren’t you; you’re writing things up on the board and even if the one is struggling a bit, then we would, we tried, you have to be careful not to take over each other’s little roles, but we did just support each other.’

### Theme 4: becoming a better practitioner

After taking part in RAFT, tutors described changes in their everyday interactions with patients, including being equipped to support patients’ self-management. They valued CBA and their increased confidence to discuss fatigue and felt that their experience ‘has certainly improved me, definitely, as a therapist’ (INT10).

#### Working with the whole person

Tutors discussed heightened awareness of looking at the whole individual and contextual factors impacting on patients’ health and not focusing solely on a set of symptoms.

INT13: “I feel I’m a better practitioner; I feel I’m more compassionate and empathetic. I think I look at them much more holistically as opposed to ‘Right, what drug can I throw at them now?’.”

#### Knowing how to draw things out, sit back and listen

Tutors contrasted CBA to support self-management in their clinical practice, with their previous ways of working.

INT6: ‘as nurses, you tend to often want to give the answer all the time and give advice and it’s very nursey to do that, but it’s learning when to listen and stand back and try and get the patients to find the answers more rather than you delivering the answers to them.’

#### Confident talking about fatigue

Tutors noted an increased confidence to discuss fatigue because they had acquired ideas, skills and tools for supporting patients.

INT7: ‘used them [activity diaries] a lot more in practice now with people, which is good; I feel a lot more happy to talk about fatigue and that with patients on a one-to-one session now.’

INT12: ‘I’ve used some of those concepts [CBA] a lot in clinic because I feel that it’s quite useful even in a few sentences.’

### Theme 5: pragmatic and flexible

Tutors expressed enthusiasm for the intervention, professional fulfilment from seeing how patients can benefit, and an interest in delivering it in clinical practice. However, they identified generic and local issues likely to impact implementation. These include sustainable training models and the importance of support from management and clinical colleagues, who need to know ‘just how you would fit it in with the other things that are going on’ (INT2). A pragmatic and flexible approach would be necessary because ‘if it’s over-prescriptive, it won’t happen, because the constraints of the NHS will just bury it’ (INT13).

#### Adapting training and support

Tutors acknowledged that 4 days of central training is not a feasible model to roll out. One option was new tutors observing the intervention being delivered by experienced tutors, either in a live setting or filmed. However, there was a strong sense that some face-to-face training with role play would be important. Tutors also stressed the need for clinical supervision to ensure fidelity to the process.

FG7: ‘trying to put those words into something that made sense to me as a non-psychologist, so having something like that [DVD], a kind of more visual thing to use alongside it [manual] I think would be really useful.’

INT10: ‘you’d want to be supervised by people who’ve got experience in it and if there aren’t people with experience in it, then a support network amongst each other.’

#### Buy-in from managers and clinical colleagues

Local roll out would typically require a business case from managers and support from clinical leads. To facilitate buy-in, tutors acknowledged the need to be flexible about who delivers the intervention. There was also enthusiasm about the possibility of delivery to other patient groups. Although tutors could envisage some changes to delivery, they did not think that the number of sessions and the topics covered could be adapted.

INT10: ‘because we’re Band 7s, they’d probably put lower bands in.’

INT12: ‘I just want to roll it out for everybody now, and I want to have CTD [connective tissue disease] groups as well as the RA groups and general arthritis groups, that would be good, and also fibromyalgia patients.’

INT11: ‘it’s difficult to think about things that could maybe be cut out to make it quicker because I think actually the benefit of it has been that all of the areas have been covered.’

## Discussion

Tutors were enthusiastic about being involved in the trial of a novel fatigue intervention, and they maintained a high level of commitment throughout the RAFT study. Although the process of becoming familiar with the material and gaining confidence in delivering it was challenging, tutors described a rewarding experience and a sense of professional development and fulfilment.

The interview and focus group findings support two key ideas about skills training and its translation into clinical practice. The first idea is the importance of role play, a method of simulation used commonly to teach communication skills [24]. In RAFT central training, role play was used in two ways. There was ‘role reversal’ with tutors taking on the role of patients to develop insight into what the group dynamics might feel like from a patient’s perspective. There was also ‘role training’, a form of role play where tutors practise the skills that can help them to become more expert in their professional role. The second idea is the importance of clinical supervision. There is evidence from a randomized controlled trial with clinical nurse specialists that although communication skills-training enhanced skills, without subsequent clinical supervision it had little effect on clinical practice [25]. Previous research in palliative care and rheumatology identified clinical supervision as a significant learning opportunity and way of consolidating and transferring newly acquired skills into clinical practice [18, 26].

During RAFT, intervention fidelity was addressed by having an independent clinical psychologist observe sessions at each site and complete a fidelity template to record the use of CBA, fatigue material from the manual, and group management techniques. It is inevitable that interventions will be adapted to local settings once they are introduced into clinical practice. Influential factors are likely to include the quality of delivery; and support, or lack of, from clinical managers. However, as intervention packaging, training and fidelity assessment have all been identified as crucial to the implementation of effective interventions in health care, it would be helpful to have a process for reporting this information [27]. Specifically, assessment should address delivery style, content, duration and coverage, and dose [28]. Exploration of implementation fidelity alongside outcome data would enable local rheumatology teams to assess quality and researchers to understand the circumstances in which a complex, self-management intervention can be effective.

Tutors’ perceptions that training and delivery benefitted their wider clinical practice highlights the potential of upskilling existing members of clinical teams to provide self-management and low-level psychological support to patients. A study with OTs and physiotherapists who trained and delivered a fatigue self-management programme in multiple sclerosis concluded that the intervention benefitted the health-care professionals involved because it expanded their clinical practice and added value to the health services they provided [29]. Evidence from other long-term conditions, such as diabetes, cardiac rehabilitation, OA-related pain and depression in long-term physical health conditions, has found that the incorporation of psychological skills into the nursing role is viewed positively by both nurses and patients [30–32]. Investment in training team members is important because the evidence is growing that CBA is clinically effective and can save costs [33–36].

Looking ahead, there was widespread support for delivery of the intervention in clinical practice, and two RAFT sites continue to offer the intervention as a clinical service. Implementation would mean training and supporting new tutors in an efficient and effective way. Tutors valued their 4 days of central training but believed that it would not be a feasible model outside the confines of a clinical trial. There is potential for developing on-line training resources and webinars, alongside some face-to-face training with the opportunity to role play key aspects of the intervention and obtain feedback from a trainer. Implementation will also require engagement with stakeholders with insights into policy drivers, such as the Academic Health Science Networks (AHSNs), which are regional bodies established by the NHS to get health innovations into practice [37].

### Strengths and limitations

This study explored tutors’ experiences and views in relationship to training, delivery and potential roll out of a novel fatigue intervention. It is a strength of the study that almost all the tutors in RAFT took part in an interview and/or a focus group; therefore, the data presented capture a wide range of views and experiences. Given the limited understanding of what might help or hinder the translation of a successful trial intervention into clinical practice, it is a strength of the study that future roll out of the intervention will be underpinned by practical approaches based on the combined perspectives of the nurses and OTs who delivered it across seven hospitals. The rigour of these findings is strengthened by having multiple co-applicants analyse the data independently, before reaching a consensus.

Data were collected after tutors had stopped delivering the intervention. This is both a strength and a limitation. On the one hand, tutors had time to reflect on their experiences and the extent to which their experience of the RAFT study continued to impact on their wider clinical practice. On the other hand, they were recalling their experiences of events that had happened a while ago. This is particularly the case for the central training, which had taken place >2 years previously. It is possible that tutors’ responses were influenced by E.D. (interviews and focus group) and S.H. (focus group) collecting the data, because the tutors met both during the central training and were informed about their involvement in the design of the intervention and the study.

### Conclusions

The RAFT randomized controlled trial has established that a manualized, group-based self-management intervention reduces fatigue impact at 6 months and 2 years. However, the implementation of evidence-based interventions in clinical practice is a challenge. This study can facilitate roll out by providing insights to inform the future training that will underpin intervention delivery and ensure its sustainability. This is likely to include a blend of on-line and face-to-face learning, comprising session demonstrations and role play with feedback from trainers. The study has also highlighted the wider benefits of upskilling members of the rheumatology team to provide self-management support to a range of patients. This additional gain could help to secure buy-in from managers and clinical colleagues, and thereby facilitate implementation. Finally, flexibility about which team members train as tutors and exploration of the potential for delivering the intervention to other patient groups with inflammatory rheumatic diseases could contribute to successful roll out.

*Funding statement*: This work was supported by the Health Technology Assessment (HTA) programme of the National Institute for Health Research (NIHR) (reference: 11/112/01). This report presents independent research commissioned by the NIHR. The views and opinions expressed by authors in this publication are those of the authors and do not necessarily reflect those of the National Health Service (NHS), the NIHR, NIHR Evaluation, Trials and Studies Coordinating Centre, the HTA programme or the Department of Health.

*Disclosure statement*: The authors declare no conflicts of interest.

## Supplementary Material

rkz032_Supplementary_Materials
