# Supplementary material for: Training and delivery of a novel fatigue intervention: a qualitative study of rheumatology health-care professionals’ experiences
Source: Rheumatol Adv Pract. 2019 Aug 27;3(2):rkz032. doi: 10.1093/rap/rkz032 (PMC6755488; doi:10.1093/rap/rkz032)
Supplement: rkz032_Supplementary_Materials [file rkz032_supplementary_materials.docx]

Supplementary materials

**RAFT intervention overview**

| **Wk** | **1^st^ hour** | **Supporting materials*** | **2^nd^ hour*** | | |
| --- | --- | --- | --- | --- | --- |
| 1 | Course purpose and expectations  Ground rules:  Commitment, confidentiality, homework  Validating fatigue: Share & discuss fatigue  experiences (difference from flare)  Self-management strategies, struggles and  difficulty of changing habits | H: Setting our course (groups’ ideas) | Energy management  -Boom & bust behaviour  -Rewards/pitfalls of this  -Prioritise, pace, plan,  -Choice is possible  H: Achieving balance  H: Activity cycling  T: Activity/rest diaries | | |
| 2 | What are your priorities for change, that  would 🡹QoL?  What are your drainers and energisers? | T: Wheel of life (priority areas) | Goal setting (two groups)  -Short/long-term goals  -Use peer group for ideas | | |
| 3 | Self-sabotage on the course  Sleep and rest:  Hours needed? Quality v quantity  Sleep hygiene strategies | H: Best ways of self-sabotage  H: Getting a better night’s sleep  T: Sleep diary (if needed) | Goal-setting review  Successes/barriers  New goals | | |
| 4 | Stress and relaxation  Personal stressors, bodily reactions  Relaxation rationale and techniques | H: Effects of stress  H: Relaxation practice guide  T: Relaxation CD | Goal-setting review  Successes/barriers  New goals | | |
| 5 | Assertiveness and communication  Passive, manipulative, assertive?  Other people’s reactions to these?  Communicating your needs | M: Cartoon examples  H: Saying ‘No’ | Goal-setting review  Successes/barriers  New goals | | |
| 6 | Review self-help tools  What have you learnt?  Review each topic  Dealing with setbacks – what could you do?  Negative self-talk, automatic thoughts,  rumination | M: Fatigue pit: Falling in/digging out  H: The pit  H: Coping with setbacks | Goal-setting review  Successes/barriers  New goals | | |
| 14 | Review last 8 wks;  Skills; dealing with setbacks;  New goals | M: Islands: Were on a Desert island (passive)  looking at the Mainland (100% health,ie  unrealistic). Now on Adaptive Coping  Island (realistic) | |  |  |

* H = Handouts, M = Metaphor, T = Tools

Reproduced from Hewlett S, Ambler N, Almeida C, Blair PS, Choy E, Dures E, Hammond A, Hollingworth W, Kirwan J, Plummer Z, Rooke C, Thorn J, Tomkinson K, Pollock J: Protocol for a randomised controlled trial for Reducing Arthritis Fatigue by clinical Teams (RAFT) using cognitive–behavioural approaches; BMJ Open 2015;5:e009061. doi:10.1136/bmjopen-2015-009061; an Open Access article distributed in accordance with the terms of the Creative Commons Attribution (CC BY 4.0) license, which permits others to distribute, remix, adapt and build upon the work, for commercial use, provided the original work is properly cited. See: <https://creativecommons.org/licenses/by-nc/4.0/>:

**RAFT Tutor interview schedule**

Introduction

This is an opportunity to discuss your experience of RAFT. Hearing about your experiences will help us to understand the practicalities, challenges and benefits of training nurses and allied health professionals to deliver the programme. As well as telling us about this research trial, your views and ideas will inform how RAFT will be rolled out in the future.

Prior to RAFT

Please tell me about:

I. Your reason(s) for deciding to take part in RAFT

II. Any previous experience of working with groups

III. Any previous experience of using cognitive-behavioural techniques

IV. Any other relevant experience (e.g. training in motivational interviewing)

Training

We would like hear your thoughts on the 4 day training that you did in Bristol:

I. The content

II. The structure

III. Your experience

IV. Would you suggest any changes?

V. How did you feel about delivering RAFT after completing the 4 day training?

VI. How did you feel about the idea of delivering RAFT after doing your first practice run?

Delivery

We would like hear your thoughts on delivering the 4 cohorts:

I. Practical challenges

II. Personal/professional challenges

III. Co-tutoring

IV. Were there particular sessions or aspects of RAFT that you found problematic or did not like?

V. Clinical supervision

a. Was this a helpful part of the process? If so, how?

b. Were there any particular issues that you sought support for?

c. Did the nature of clinical supervision change over time?

VI. Did delivery of each cohort feel different? If so, how?

Impact on wider clinical practice

I. Has taking part in RAFT had any impact on your wider clinical practice?

II. Do you perceive any benefits to patients? / Do you perceive any drawbacks for patients?

III. Do you perceive any benefits for your professional development? / Do you perceive any drawbacks for your professional development?

In the future

Thinking about how we might roll out RAFT in the future:

I. Are you and your wider team likely to support the delivery of RAFT in the future?

II. Do you know whether you could access clinical supervision locally?

III. Do you think training needs to be face-to-face? In a group? Would DVDs be useful?

IV. Would you recommend changes to the manual? If so, can you describe them?

Close

Are there any other aspects of your experience or thoughts about RAFT in the future that you would like to tell us about? Thank you for your time
